# Changing Hearts and Minds in the Equestrian World One Behaviour at a Time

**DOI:** 10.3390/ani13040748

**Published:** 2023-02-19

**Authors:** Inga A. Wolframm, Janet Douglas, Gemma Pearson

**Affiliations:** 1 Applied Research Centre, Van Hall Larenstein University of Applied Sciences, Larensteinselaan 26-A, 6882 CT Velp, The Netherlands; 2 World Horse Welfare, Anne Colvin House, Snetterton, Norwich NR16 2LR, UK; 3 The Horse Trust, Slad Lane, Princes Risborough, Buckinghamshire HP27 0PP, UK; 4 Easter Bush Campus, The University of Edinburgh, Midlothian EH25 9RG, UK

**Keywords:** behaviour change wheel, COM-B, equestrian sport, equestrianism, human behaviour change, learning theory, social licence to operate

## Abstract

**Simple Summary:**

Equestrianism is currently facing a range of pressing challenges. These challenges are largely based on evolving attitudes to ethics and equine wellbeing and affect the sport’s social licence to operate (i.e., its public acceptability). It is likely that trends within society, features specific to the equestrian sector, and aspects of human nature have all contributed to the current situation. If equestrianism is to flourish, it is evident that much needs to change, not the least, human behaviour. There are established frameworks for explaining and effecting human behaviour change that have been scientifically validated and are rooted in practice. These frameworks, such as the COM-B model and the Behaviour Change Wheel by Michie et al., could be of practical value for developing and implementing equine welfare strategies. The current review summarises the theory that underpins some behaviour change frameworks and provides a practical, step-by-step approach to designing an effective behaviour change intervention. A real-world example is also provided. This is based on retrospective analysis of an intervention strategy that aimed to increase the use of learning theory in (educational) veterinary practice. In our opinion, incorporating effective behaviour change interventions into any equine welfare improvement strategy may help to safeguard the future of equestrianism.

**Abstract:**

Equestrianism is currently facing a range of pressing challenges. These challenges, which are largely based on evolving attitudes to ethics and equine wellbeing, have consequences for the sport’s social licence to operate. The factors that may have contributed to the current situation include overarching societal trends, specific aspects of the equestrian sector, and factors rooted in human nature. If equestrianism is to flourish, it is evident that much needs to change, not the least, human behaviour. To this end, using established behaviour change frameworks that have been scientifically validated and are rooted in practice—most notably, Michie et al.’s COM-B model and Behaviour Change Wheel—could be of practical value for developing and implementing equine welfare strategies. This review summarises the theoretical underpinnings of some behaviour change frameworks and provides a practical, step-by-step approach to designing an effective behaviour change intervention. A real-world example is provided through the retrospective analysis of an intervention strategy that aimed to increase the use of learning theory in (educational) veterinary practice. We contend that the incorporation of effective behaviour change interventions into any equine welfare improvement strategy may help to safeguard the future of equestrianism.

## 1. Introduction

### 1.1. Changing Attitudes in and towards Equestrianism

Equestrian sports and recreational activities are increasingly under societal pressure to review their approach to safeguarding equine welfare [1]. As incidents of equine mismanagement and abuse at high-profile events [2,3,4] and popular tourist attractions (e.g., [5]) grace the virtual front pages of media outlets worldwide, it is not just animal activists and welfare organisations that are voicing their concerns. The general public is also beginning to take a more critical look at an activity that, until now, has mostly been viewed favourably [6]. A combination of changing attitudes towards animal welfare [7], increasing (bio)ethical awareness [8], advances in communication technologies [9,10], and the pervasiveness of social media [11,12] all contribute towards a shift in society’s view of equestrian activities. Practices that were once considered acceptable are now being questioned by individuals and organisations with no direct links to equestrianism [1,13,14]. For those who support equestrianism, this necessitates the examination of current practices and—if they wish to protect the future of their sport—a willingness to change [15].

### 1.2. Bone of Contention or a Blessing in Disguise? The Social Licence to Operate

The concept of “Social Licence to Operate” (SLO) and the question of how to integrate pressures from outside the equestrian sector with the internal reluctance to change have become important topics of consideration at the highest levels of equestrianism [16]. As outlined by Douglas et al. [15], an SLO is an “intangible, implicit agreement between the public and an industry/group”. In order to conduct its activities with a minimum degree of formal restriction, an industry or sector must ensure that it gains and maintains the acceptance of the society in which it operates. The implications of failing to do so have become evident in recent years. For example, the decision to remove the horse riding phase from Modern Pentathlon and replace it with an obstacle course for human athletes has been seen by many as a direct consequence of the distressing scenes at the 2020 Tokyo Olympics [16], where equine welfare was compromised due a combination of factors, including the actions of both human athletes and officials [2]. The International Modern Pentathlon Union called the change a historic move aimed at modernising the sport and boosting its suitability for future Olympics [17]. Yet, behind this rhetoric, the move is indicative of the power of public opinion and what may be to come if equestrianism fails to take decisive action to protect equine welfare.

### 1.3. The Need for a Welfare Strategy

In a direct response to a perceived threat to equestrianism’s SLO, the International Equestrian Federation (FEI) recently established the Equine Ethics and Wellbeing Commission, a group that has been tasked with developing independent, objective advice and making recommendations to the FEI on matters relating to ethics and wellbeing in equestrian sport [18]. As outlined by Ingmar de Vos, President of the FEI: “[…] in an ever-changing society, where perceptions shift and norms evolve at an increasingly fast pace, the FEI must address these concerns and criticisms from society and within equestrian circles in a clear and transparent manner” [19]. It seems clear that in order to safeguard both equine welfare and the sustainability of equestrianism as a whole, the development of a coherent, evidence-based welfare strategy is imperative.

## 2. The Future of Equestrianism in the Context of Human Behaviour Change

### 2.1. Strategy First, Implementation Second

The welfare of horses is ultimately dependent on human behaviour. Therefore, when one is considering the design and implementation of an equine welfare strategy, some general questions should be addressed. For example: What does the strategy entail? Are there any specific practices or behaviours that need to be changed, kept the same, or enhanced? How do any new practices or behaviours differ from current ones and what barriers might prevent these new practices from being adopted? Who are the key stakeholders that either need to perform the new behaviours or are instrumental in facilitating or preventing them? Additionally, how do we know whether an implementation has been successfully executed?

The implementation of evidence-based practices can be fraught with challenges (e.g., [19,20,21]), and approximately two-thirds of initiatives may fail to achieve their goal [22]. Experiences involving behaviour change initiatives in complex and dynamic industries such as the health sector [23,24], energy consumption [25], efforts to halt climate change (e.g., [26]), and zoo conservation programs [27] demonstrate that supplanting undesirable or counterproductive behaviours with more desirable ‘target’ behaviours can be a challenge, even if there is sufficient scientific evidence to support the need for change. 

In any situation, human beings have a range of behavioural options. These are governed by a combination of internal processes (e.g., individual resources and conscious and sub-conscious mental processes) and external factors (e.g., contextual cues and environmental resources) [23,28]. The likelihood of a person enacting each of their behavioural options at any particular time and in any particular context is known as the ‘behavioural potential’ of that option [23]. In any set of conditions (time, context, etc.), an individual is likely to enact the behavioural option with the highest behavioural potential. As a result, whenever a particular set of conditions occurs regularly, that behaviour will become the dominant behavioural response [29,30]. For any new behaviour to supersede a previously conditioned dominant behaviour, the new behaviour must hold a greater behavioural potential than the original dominant behaviour [29,30]. Moreover, individuals tend to overvalue short-term rewards and underestimate long-term benefits [20], making behaviour change particularly complex in situations where the value assigned to future outcomes is uncertain [31].

### 2.2. The COM-B Model as a Starting Point for Eliciting Behaviour Change

**Figure 1:**
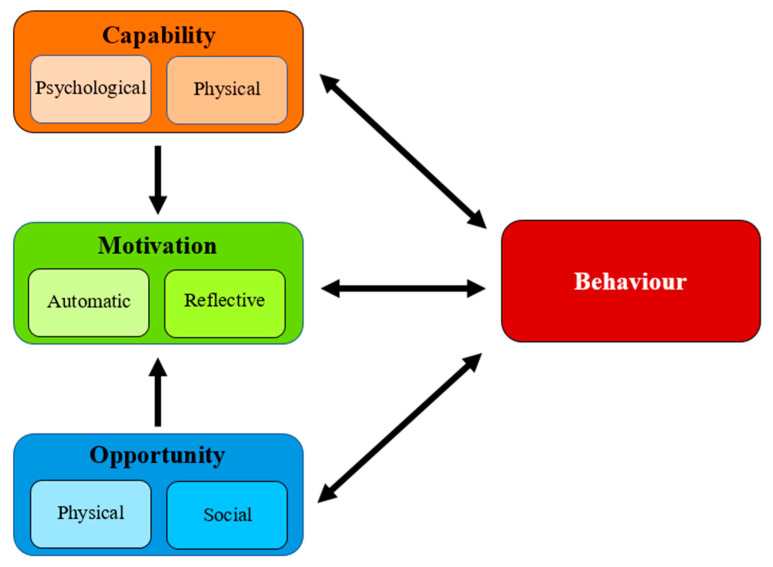
The COM-B model, showing the three sources of behaviour and their sub-components. Reproduced with permission [33] from Michie, Atkins, and West (2014) [33].

In 2011, Michie et al. [32,33] developed the COM-B model of behaviour change. This model serves to both explain behaviour and provide the foundation for behaviour change. It is also sufficiently broad to encompass most, if not all, behaviour settings. According to Michie et al. [32], behaviour—whether it is deliberate or unintentional—depends on the interaction of three sources: capability, opportunity, and motivation (Figure 1). 

“Capability” covers whether an individual or organisation possesses the psychological or physical capability (i.e., knowledge or skills, respectively) necessary to execute the desired behaviour [32,34,35]. “Opportunity” describes the physical and/or social environment which may, or may not, be supportive of the behavioural change and/or conducive to the individual or organisation engaging in the target behaviour. 

“Motivation” refers to mental processes of the individual (or organisation) that initiate and direct behaviour. Such processes can be ‘automatic’ (i.e., affected by habit, desire, instinct, natural drive, etc.) or they may be ‘reflective’ (i.e., determined through conscious thought such as goal setting or planning) [32,33,34]. 

While capability, opportunity, and motivation all influence behaviour, the levels of capability and opportunity are considered to be the drivers of motivation, with motivation ultimately determining whether a behaviour is executed. Simply put, the more capable the individuals are, and the more resources and support there are at their disposal, the more motivated they will be to execute a behaviour [32,33,35,36]. Nevertheless, each situation is different in terms of the impact that each of the three factors has on facilitating or preventing behaviour change [32,33]. Therefore, when one is considering behaviour change in any industry or activity, it is vital to determine the extent to which each of the three factors may influence the execution of the desired target behaviour. 

Applying this to equestrianism, we need to establish what might stand in the way of changing equestrian practices. Is it a lack of knowledge or skill? Does the physical environment or social context prohibit the execution of the behaviour? Or do ingrained habits, instincts, or even a lack of planning prevent meaningful change? A thorough analysis aimed at determining any potential barriers to the desired target behaviours is an essential first step towards mapping out an effective behaviour change strategy [37,38]. 

### 2.3. The Behaviour Change Wheel as a Guide for Intervention Design

**Figure 2:**
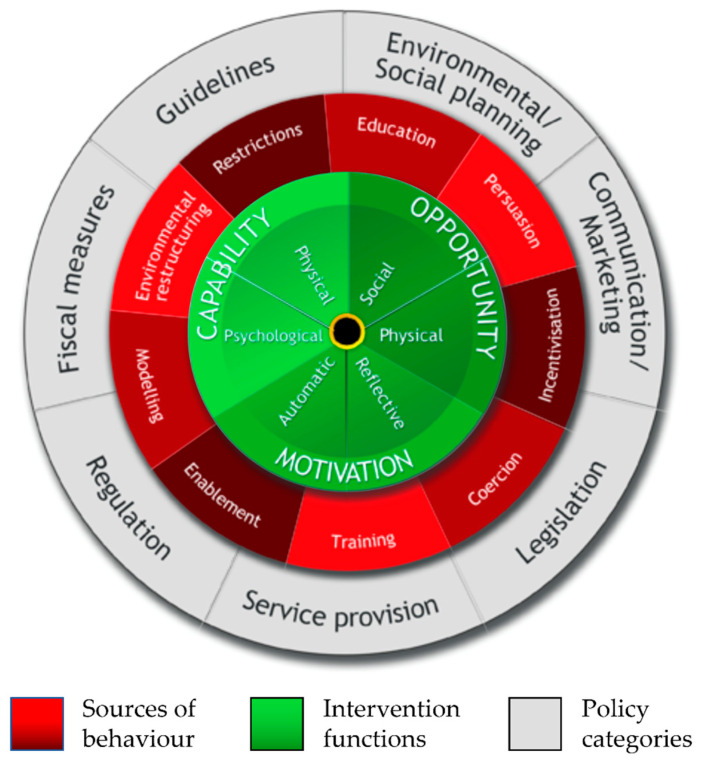
The original Behaviour Change Wheel, showing the three sources of behaviour, the nine intervention functions, and the seven policy categories. Reproduced with permission [33] from Michie, Atkins, and West (2014) [33].

When Michie et al. [32,33] developed the COM-B model, they also defined nine ‘intervention functions’ and seven ‘policy categories’. Taken together, these components form the Behaviour Change Wheel (Figure 2).

The intervention functions describe how the behaviour might be changed by targeting the specific sources of behaviour. Depending on the circumstances and individuals involved, insufficient knowledge (“capability psychological”), a non-existent or weak social support network (“opportunity social”), a lack of finances (“opportunity physical”), or an ingrained habit (“motivation automatic”) might prevent the adoption of a better, safer, more welfare-orientated behaviour. These barriers may act alone or in combination. 

When one is designing implementation strategies, it is critical, therefore, to first of all determine where the barriers might lie. Using the COM-B model as a guideline, the relevant questions to determine these barriers might be: Does the group whose behaviour we are trying to change have sufficient knowledge at the theoretical or practical level? Do they need different or additional skill sets? Does their physical environment lend itself to the target behaviour being performed? Do they have the necessary resources (e.g., time and money)? Is their social network supportive of the change? Are there deeply ingrained habits or instincts that are contrary to the target behaviour? Has attention been paid to structured planning or goal setting? Depending on the answers, it becomes possible to determine which intervention function(s) are likely to be most effective for any particular situation. As outlined in Table 1, the nine intervention functions all map onto (i.e., influence) one or more of the sources of behaviour in the COM-B model and provide a pragmatic starting point from which to design interventions. (Some researchers might prefer a more detailed approach to developing behaviour change interventions. They are advised to consult the Theoretical Domains Framework (TDF, REF 10; 29 BCW book), which was developed from 83 behavioural change theories, with overlapping theoretical constructs. The resulting 14 domains provide additional guidelines to develop behavioural analyses and interventions).

The seven ‘policy categories’ support the interventions. So, for example, an intervention strategy might involve the intervention functions ‘education’ and ‘regulation’, which might, in turn, be implemented by drawing on the policy categories of ‘communication/marketing’, ‘development of guidelines’, and/or ‘legislation’. 

### 2.4. Multi-Stakeholder, Multifaceted Behavioural Change Interventions

The Behaviour Change Wheel presents us with an opportunity to revise our approach to improving equine welfare. In addition to continuing to push for more research and the communication of factual information, we should focus on the implementation of behaviour change strategies that include reflective motivational components and that encourage engagement and interaction between the various stakeholders at different levels of the industry. A multifaceted, multi-stakeholder approach is likely to yield the greatest chance of success if it is aimed at the entire support network (i.e., the equestrian community), rather than focusing solely on the individual, and should be directed at all levels within equestrianism, from the grass roots level to the top level of elite sport. 

**Figure 3:**
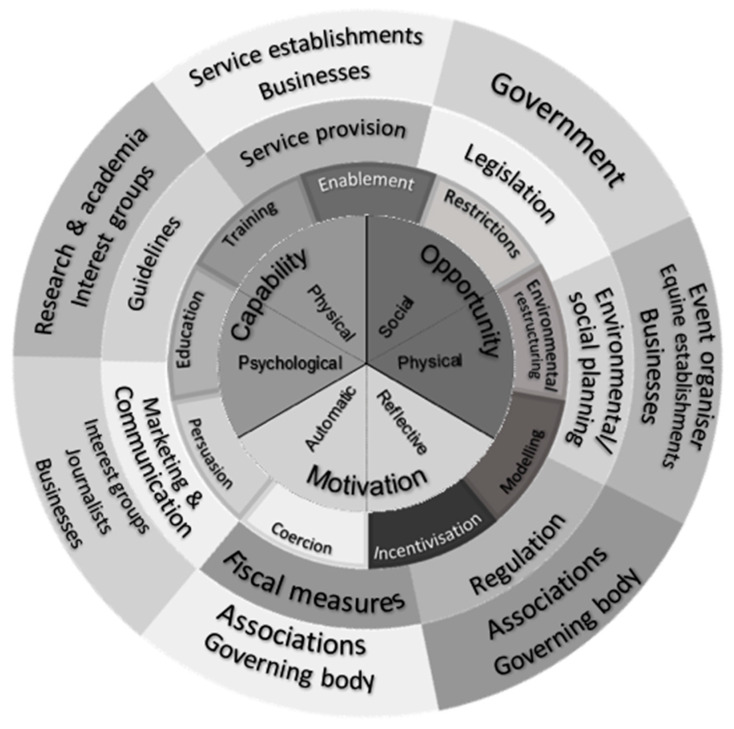
Original Behaviour Change Wheel [33] populated with stakeholders relevant to equestrianism.

Figure 2 shows the original Behaviour Change Wheel, as published by Michie et al. [32,33], in which they identified overarching policy categories for each of the intervention functions. However, if the Behaviour Change Wheel is to be used in the context of equestrianism, we advocate supplementing—or, if appropriate, replacing—these policy categories with the equestrian stakeholders that may be involved in the implementation of the interventions (Figure 3). By assigning behaviour change interventions to relevant stakeholders, these individuals/organisations become part of the behaviour change system, creating engagement and commitment within the wider equestrian community right from the start, thereby increasing the chances of sustained success [39]. 

### 2.5. Effectiveness and Sustainability of Behaviour Change

It has been well documented that most people find it difficult to change their behaviour [40,41,42,43], even if they have succeeded at identifying their own personal barriers to change [40]. Moreover, while behaviour can be modified through interventions [44,45], the intervention effects often diminish over time [23]. This means that the maintenance of the newly acquired behaviour can present additional challenges [41,46]. It is therefore important to design interventions that maximise both the effectiveness and longevity of the behaviour change. 

In terms of longevity, research into pro-environmental behaviour change has shown that interventions that are embedded in community structures and social networks, and those that take into account the wider systemic and institutional implications of the intervention, are more likely to lead to more sustainable results than those that do not incorporate these features [47,48]. Behaviour intervention research in other fields has shown that targeting the social environment (i.e., “opportunity”) is more effective than merely focusing on the individual [42]. For example, interventions that include an interactive ‘social’ component are more valuable than those that just provide facts [49,50,51]. 

Similarly, interventions that focus solely on capability building (e.g., providing advice or educational measures) are less effective than those that include motivational components such as goal setting [52,53,54,55]), self-monitoring [53,54,55], the provision of feedback on performance [53,56], or motivational interviewing [55,57]. These findings further support the general premise of the COM-B model: while knowledge and skills (“capability”) certainly play a part in influencing behaviour, it is the motivational factors, such as automation and reflection, that are the main drivers behind behavioural change. These are important lessons for the equine sector, which traditionally, has focused on knowledge transfer and training as the most desirable tools to improve equine welfare (e.g., [58,59,60,61,62]).

## 3. Behaviour Change in Practice

In this section, we outline a step-by-step approach to designing a behaviour change intervention strategy, based on the Behaviour Change Wheel [33]. More in-depth information surrounding the Behaviour Change Wheel can be found in Michie et al.’s original work [32,33,34,35,36]

**Step 1:** Identify the problem behaviour.

**Step 2:** Determine the desired target behaviour; this behaviour should have been shown (or at least be theorised) to lead to better outcomes than the problem behaviour does. 

**Step 3:** Identify internal and/or external barriers that might prevent the execution of the target behaviour by asking questions that relate to each of the three factors of the COM-B model and their sub-components. This is best accomplished by thinking of all the different aspects related to capability (psychological and/or physical), opportunity (physical and/or social), and motivation (automatic and/or reflective) that an individual or organisation might need to have access to or possess in order to perform the target behaviour [32,33]. 

**Step 4:** Develop a behaviour change strategy that comprises intervention functions (see Table 1) that specifically target the barriers determined in Step 3 (while bearing in mind the target group, existing amenities, costs, ease of execution, etc.) [33]. Note that some potentially effective interventions may not be feasible for practical, financial, or ethical reasons. 

**Step 5:** Designate the intervention owner by determining which stakeholder(s) is/are best suited to implement one or more interventions. Develop the necessary tools/media and means of delivery.

**Step 6:** Determine the order in which the interventions should be rolled out and a timeline for the intervention delivery. Note that certain interventions might rely on others being completed or ongoing and that, if the intervention design team is small, it may not be possible to initiate all of the interventions simultaneously. Prioritisation may, therefore, be key to the successful implementation of the strategy. 

**Step 7:** Establish parameters that support the assessment of behaviour prior to and after implementation of the intervention. Use these to measure its effectiveness. 

## 4. Putting Theory into Practice: Retrospective Analysis of an Intervention Aimed at Incorporating Learning Theory into Veterinary Practice

### 4.1. Background and Context

Veterinary care is one of the most dangerous professions in the world, and equine veterinarians typically sustain multiple injuries during their working lifetime [63,64]. Unwanted and potentially unsafe equine behaviours range from not standing still or being bargy (pushy) to more dangerous behaviours such as rearing, striking, or kicking with a hind foot. When dealing with difficult horses, veterinarians often rely on physical restraint [65] (e.g., holding up a leg or using a twitch) and/or sedation [63]. However, sedation and physical restraint are not a panacea. In a survey of 620 British equine veterinarians [63], 37% of 1,142 injuries that required treatment and/or resulted in time off work occurred while the horse was sedated, and an additional 30% occurred when the horse was being ‘controlled’ using another form of restraint (59% twitch; 23% stocks). Moreover, even if restraint is utilised successfully on one occasion, the horse is likely to develop a negative association with that scenario [64] and be more likely to react adversely the next time [66,67].

A potentially more effective and safer alternative is to train the horse to accept the procedure using the principles of learning theory as part of a behaviour modification plan. If this is performed successfully, the horse ceases to recognise veterinary care as a threat, and so becomes compliant [66,67]. One author (GP) initiated a behaviour change intervention on this topic among veterinary students as representatives of the future generation of veterinary practitioners.

### 4.2. Step-by-Step Approach to Designing a Behaviour Intervention

***Step 1: Define the problem behaviour***

In this case study, the veterinarians’ use of traditional means of restraint constitutes the problem behaviour.

***Step 2: Define the target behaviour***

The target behaviour is defined as encouraging veterinary students to employ the principles of learning theory to retrain equine patients that were previously perceived as ‘difficult’ or non-compliant during treatment.

***Step 3: Determine barriers to performance of the target behaviour***

Relevant barriers were categorised using the COM-B model (summarised in Table 2).

*Capability*

Psychological: In the survey of 168 equine veterinarians conducted by Pearson et al. [65], the majority of the respondents reported that they understood how horses learn and were able to apply that knowledge. Yet, when they were tested on the topic, they performed poorly. Moreover, when they were asked what could be done to reduce the injury rates, these individuals focused exclusively on methods of physical or chemical restraint. None of the responses mentioned the application of learning theory or addressed the fact that equine-related accidents generally occur as a result of a horse’s behavioural responses to a potential threat (stressor). The apparent gap between the perceived and actual knowledge regarding equine learning theory may be viewed as a barrier to veterinarians performing the target behaviour.

Physical: Pearson et al. [68] demonstrated considerable variability among veterinarians in their ability to observe behavioural indicators of stress in horses undergoing veterinary care. Veterinarians who miss subtle stress responses are more likely to handle horses inappropriately. An inability to identify stress responses may be considered as a barrier in terms of ‘physical capability’ (i.e., skills). Furthermore, at the time of this intervention, veterinary schools traditionally focused their equine handling classes on physical restraint, with learning theory and behaviour modification plans aimed at facilitating patient compliance being rare [69]. Some schools even emphasised that the use of physical restraint (e.g., nose and neck twitches) was important for safety. Undergraduate students, therefore, did not develop the skills (physical capabilities) required for success.

*Opportunity*

Physical: Veterinarians who work with horses are often under pressure to complete their work in a timely manner [70]. The most common reason encountered when resisting the use of behaviour modification in their patients is that they ‘do not have time’ [65].

Social: Data from a number of countries suggest that the risk of injury to equine veterinarians is not only high but, to some extent, expected [63,71,72]. This suggests that equine veterinarians may consider injuries to be a ‘part of the job’. Being able to handle this risk might be considered as something akin to a rite of passage, with the associated social pressure to simply ‘get on with it’ having evolved into a barrier to adopting the use of learning theory.

*Motivation*

Automatic: Until recently, veterinary schools generally focused on teaching traditional methods of restraint [69]. For young veterinarians, this would be reinforced when they entered clinical practice, where more experienced vets—who function as role models—are likely to also use these methods. The use of such techniques is, therefore, likely to become habitual in younger veterinarians.

Reflective: Veterinarians commonly state they are there to treat horses, rather than to train them [69]. This shift of responsibility from the vet (who is constantly influencing the horse’s behaviour during interactions, whether intentionally or not) [64] to the owner may be a way of justifying their actions (or lack thereof). However, veterinary graduates report that having effective animal handling skills is helpful in building relationships with their clients [70,73], who may judge veterinarians’ level of competence by their ability to handle a horse. Veterinary students may therefore strive to demonstrate competence in this area, but if they are not aware that there are safer and more equine-friendly techniques other than physical or chemical restraint, it will be almost impossible for them to reflect on a more effective way of doing things.

***Step 4: Develop behaviour change intervention(s)***

As outlined above, the barriers to veterinarians employing learning theory when they are handling horses can be identified across all three factors and all six subfactors of the COM-B model. To cover all of the barriers, an effective behaviour change intervention must, therefore, include several intervention functions. The behaviour change intervention strategy chosen by Pearson et al. [65] combined aspects of education, training, persuasion, modelling, and enablement and addressed all six sources of behaviour/potential barriers (Table 1).

The intervention involved providing a cohort of fourth year veterinary students (N = 157) with five video scenarios of horses demonstrating unwanted behaviours during veterinary care [74]. The students were asked about the factors that motivated the horses’ responses, how likely they would be to use various suggested strategies for dealing with the scenario and, if they were presented with the scenario in practice, how confident they would feel, their anticipated chances of success in completing the veterinary intervention, and the perceived risk of injury to themselves. Following this, the students attended a 45-min lecture on equine behaviour, with an emphasis on three key learning objectives:

1. To understand the processes by which horses learn (learning theory).
  - This knowledge is essential to change how the students perceive what they might do in each scenario;
  - Relevant intervention function: education.
2. To be able to develop a shaping plan (i.e., a breakdown of the final behaviour into a series of steps, each of which is easily achievable).
  - This enables student to succeed at physically implementing the techniques, with a focus on goal setting, action planning, and problem solving;
  - Relevant intervention functions: training and enablement.
3. To appreciate subtle behavioural indicators of stress in horses.
  - This allows students to adjust their plan based on how the horse responds; this is important if the students are to have the physical capability to implement these techniques, to visualise themselves doing so, and to plan and review their actions and goals;
  - Relevant intervention functions: training and enablement.

These learning objectives were supported by multiple video case examples which were played—where possible—in real time from start to finish and demonstrated by a credible source (an experienced veterinarian) [74]. These videos showed the students that these techniques are feasible and effective in real-life scenarios, while focusing on problem solving, action planning, and goal setting (relevant intervention functions: modelling and enablement). The emphasis was placed on using positive, solution-focused language to teach the students how to cope with different scenarios, rather than telling them what not to do or making negative comments about the techniques that they may have seen used by other veterinarians. The videos also demonstrated the rapid effect on the horse of the use of learning theory (relevant intervention function: persuasion).

***Step 5: Designate intervention owner and determine delivery***

**Figure 4:**
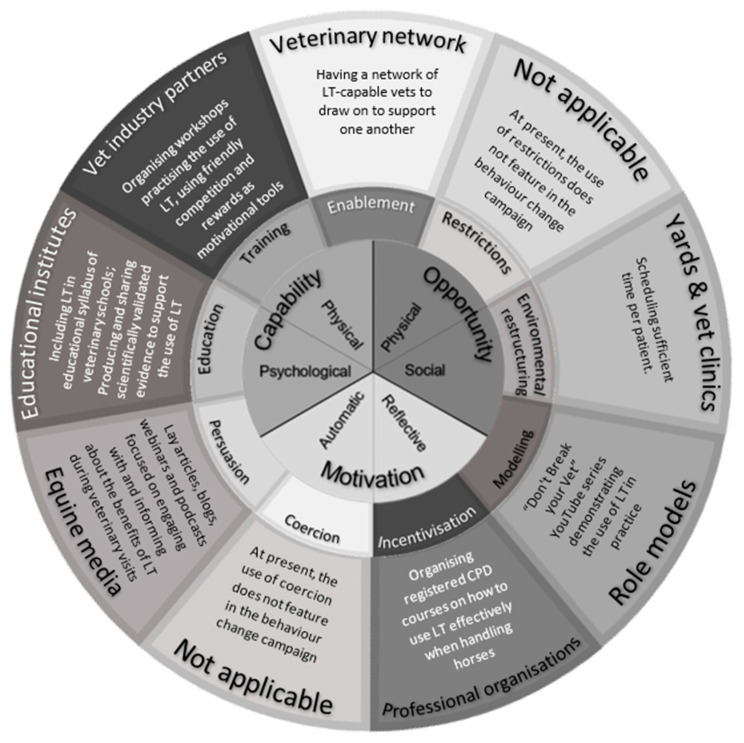
Overview of continued multi-intervention, multi-stakeholder behaviour change strategy to encourage the use of learning theory in equine veterinary practice. (LT = learning theory; CPD = continuing professional development).

The intervention owner—who also determined its delivery—was a veterinary professional working in a university teaching role (GP). (Note that when they are trying to induce behaviour change in a more complex environment, different stakeholders should be involved in both the planning and delivery stages—see Figure 4).

***Step 6: Determine timeline to roll out the intervention***

The video-based questionnaire that was distributed immediately prior to the lecture intervention (‘pre’ questionnaire; see step 4) was repeated one week after the lecture (‘post’ questionnaire) and at the end of the students’ 4-week rotation in the equine hospital (‘delayed post’ questionnaire: 7–31 weeks after the lecture). The ‘delayed post’ questionnaire was distributed at this time, rather than at a fixed time point after the lecture, because it was only during this rotation that the students would see qualified veterinarians deal with scenarios similar to those in the videos. Pearson et al. were keen to capture the students’ exposure to this clinical environment in their responses.

***Step 7: Measure intervention effectiveness***

The comparison of the ‘pre’ vs. ‘post’ and/or ‘delayed post’ questionnaires (from the 47 students who completed all three of them) showed that the behaviour change intervention led to them being significantly less likely to consider the horses in the videos as being naughty (all five scenarios) or dominant (three out of five scenarios) and significantly more likely to say that they would use learning theory to manage each scenario. The intervention was also associated with them reporting, for all five scenarios, that they would feel significantly more confident, at significantly lower risk of injury, and significantly more likely to succeed.

The students could leave comments at the end of both the ‘post’ and ‘delayed post’ questionnaires. Although we cannot be sure what the students actually did when they were presented with a non-compliant horse, these comments may indicate how their behavioural potential had changed, with several students describing how they had applied learning theory in practice. In association with the questionnaire results, these comments demonstrate the effectiveness of the intervention and the value of using the Behaviour Change Wheel as a framework for the design and implementation of a behaviour change intervention strategy. 

Below, we have grouped the students’ comments according to the most relevant COM-B source of behaviour and have highlighted the text that supports this categorisation.

**Capability (physical)**

- “This was **a very useful session**”
- “…the…lecture…**helped put me at ease and allowed me to deal a lot better** with certain situations”;
- “**I was able to utilise the techniques**…they worked extremely well”;
- “I used Gemma’s techniques…and **it worked great**!”;
- “I **was successful in giving a horse oral medicine** who, previously, would head buck”;
- “Even the quick tips…**will be so handy in practice**”;
- “I have **used some of these techniques on EMS [extra-mural studies] and they have worked really well**”;
- “**Worming the teaching ponies**—used information gathered in the lecture/videos—was **very impressed and surprised at how effective it was**”;
- “Some of the techniques demonstrated were just **brilliant ways to deal with really common scenarios**”.

**Capability (psychological)**

- “I have been working with horses since a young age in racing, eventing, and competition yards. I would have considered that I knew a reasonable bit about horse behaviour … but this tutorial **really gave food for thought**”;
- “**Learning about equine behaviour made it a lot safer** to work in the equine hospital”;
- “**Learning about equine behaviour has really helped me understand the way horses react**…and how simple it can be to teach them not to react badly”;
- “Videos **really help to understand** how to do clicker training”.

**Motivation (automatic)**

- “…**made me feel a lot more comfortable** about working with more difficult horses.”;
- “…**made me feel a lot happier** about working with some of the more difficult horses”;
- “lecture with fantastic video evidence…**highlighted some of my previous mistakes** when dealing with badly behaved horses”;
- “…this class **has made me feel much more confident** about working with these animals”.

**Motivation (reflective)**

- “**I think it will help me to be a safe and competent vet**”;
- “amazing examples **motivated me to try it out for myself**…definitely helped me administer medications to head-tossing horses in a less stressful manner, for the horse and for myself”;
- “it was amazing how much effect even a few minutes of proper handling and learning had on the horses…**it’s an incredibly important thing for us as students**”;
- “**we were wishing that [Gemma] was present** to teach us how to deal with…difficult patients;
- “some of this stuff **has definitely come in handy**”;
- “I…am quite easily intimidated by “difficult” ones but **it really helped to think about the ways you can get around it**”.

**Opportunity (social) and (physical)**

- “…the behaviour lecture was really beneficial…**a few residents and myself** used some of the techniques [from] the lecture…this worked really well in less than 10 min”;
- “I am currently seeing practice with an equine vet and **I was chatting with her about a needle-shy horse I had seen with Gemma whilst I was on ICU and she was really interested in the behaviour techniques rather than just shouting at the horse**”;
- “Would have been great to **have had time set aside** to learn how to do it on hospitalised cases”.

### 4.3. Continuation of the Intervention

The third author (GP) is involved in ongoing efforts to achieve more widespread use of learning theory in the veterinary community. Figure 4 provides a brief overview of the current behaviour change interventions that have been initiated in cooperation with different stakeholders. A number of these interventions have involved demonstrating, under first opinion veterinary practice ‘field’ conditions, how rapidly the use of the learning theory can effect change in a horse’s behaviour. Anecdotal evidence suggests that these initiatives are gradually taking effect, and research is planned to support this with scientific evidence. As discussed above, changing behaviour takes time, especially if the target behaviour is to become the primary option of the behavioural repertoire. A sustained multifaceted, multi-stakeholder intervention is therefore likely to be necessary if a target behaviour is to become embedded in practice. 

## 5. Conclusions

A combination of changing attitudes towards animal welfare, increasing (bio)ethical awareness, advances in communication technologies, and the pervasiveness of social media seems to have set in motion a shift in society’s acceptance of equestrian activities. As a result, equestrianism is under growing pressure—from both inside and outside the sector—to review its approach to safeguarding equine welfare. While it is clear that a comprehensive equine welfare strategy is required to turn the current tide, it seems equally clear that the success of any such strategy relies on the degree to which human behaviour subsequently changes in both the short- and long-term. 

This paper has demonstrated—from both theoretical and practical points of view—how one might design an effective behaviour change intervention strategy using a structured, scientifically validated, step-by-step approach that enables the definition of the problem and target behaviours, identification of any behavioural barriers that may prevent execution of the target behaviour, and design of relevant behavioural change intervention functions. Our retrospective analysis of a behavioural change intervention that targeted equine veterinary students shows how this concept—which may appear to be somewhat abstract to those who have not previously used behaviour change frameworks—can translate into real-world practice. 

The case study demonstrated what a multifaceted intervention might look like in terms of execution, as well as its potential effectiveness. It also highlighted some of the difficulties surrounding behaviour change interventions: while an intervention might initially appear to be promising, it will only truly make a difference if the resulting behaviour change proves to be sustainable in the long term and in different situations. As such, the use of validated behaviour change models such as Michie et al.’s Behaviour Change Wheel combined with a sustained, coordinated, multistakeholder effort to effect behaviour change could provide the equestrian sector with important tools in the practical implementation and application of any future welfare strategy aimed at safeguarding the future of the sport.

## Figures and Tables

**Table 1:** Mapping ^1^ of Behaviour Change Wheel intervention strategies to COM-B sources of behaviour [33].

Behaviour Change Wheel	COM-B Sources of Behaviour					
Intervention Functions	Capability		Opportunity		Motivation	
	Psychological	Physical	Physical	Social	Automatic	Reflective
**Education** Increasing knowledge or understanding						
**Training** Imparting skills						
**Persuasion** Using communication to induce positive or negative feelings or stimulate action						
**Incentivisation** Creating expectation of reward						
**Coercion** Creating expectation of punishment or cost						
**Restriction** Using rules to: (i) increase the opportunity to engage in the target behaviour or (ii) increase the target behaviour by reducing the opportunity to engage in competing behaviour						
**Environmental restructuring** Changing the physical or social context						
**Modelling** Providing an example that people can imitate or to which they may aspire						
**Enablement** Increasing means/reducing barriers to increase capability or opportunity						

^1^ Grey shading denotes the relationship between sources of behaviour and intervention functions (e.g., education influences psychological capability and reflective motivation).

**Table 2:** Summary of barriers to performance of target behaviour (veterinary students’ use of learning theory to retrain non-compliant horses).

**Capability Psychological**
Learning theory and behaviour modification plans aimed at facilitating patient compliance are not taught at veterinary schools; the practitioners’ understanding of learning theory is generally poor
**Capability physical**
Students are unable to develop relevant skills because the subject is not taught or practiced at veterinary schools; the practitioners’ ability to identify behavioural signs of stress in horses is generally poor
**Opportunity physical**
Equine veterinarians are typically under pressure to complete tasks quickly
**Opportunity social**
Equine veterinary ‘culture’ regards injury as an unavoidable occupational hazard and the ability to deal with a non-compliant horse in a physical manner as a ‘badge of honour’
**Motivation automatic**
Veterinary schools and role models focus on traditional methods of restraint
**Motivation reflective**
Reflection is not possible if they are unaware of more effective methods

## Data Availability

No new data were created or analysed in this paper. Additional details pertaining to the data presented in Section 4 ‘Putting Theory into Practice’ can be found in Pearson, G.; Reardon, R.; Keen, J.; Waran, N. Difficult Horses–Prevalence, Approaches to Management of and Understanding of How They Develop by Equine Veterinarians. Equine Vet. Educ. 2021, 33, 522–530. https://doi.org/10.1111/eve.13354.
